# Antioxidant Properties of the Methanol Extract of the Wood and Pericarp of *Caesalpinia decapetala*

**DOI:** 10.4103/0975-1483.62212

**Published:** 2010

**Authors:** CR Pawar, SJ Surana

**Affiliations:** *Department of Pharmacognosy, R.C. Patel College of Pharmacy, Shirpur, Dhule, Maharashtra, India*

**Keywords:** *Caesalpinia decapetala*, antioxidant activity, free radical scavenger

## Abstract

The antioxidant activities of the methanol extracts from the wood and pericarp of *Caesalpinia decapetala* (Roth) Alston (Caesalpiniaceae) were assessed in efforts to validate the herb. The antioxidant activity of the plant has been studied using its ability to scavenger DPPH, superoxide radicals, and nitric oxide radical along with its ability to inhibit lipid peroxidation. The antioxidant activity and phenolic content of the pericarp as determined by the DPPH, superoxide radical, nitric oxide radical, total phenols, the flavonoids, and total flavonols were higher than that of the wood. Analysis of plant extracts revealed a high amount of polyphenols and flavonoids suggesting a possible role of these phytoconstituents in the antioxidant property. Moreover, the results were observed in a concentration and dose dependent manner. Studies clearly indicate that the *C. decapetala* has significant antioxidant activity.

## INTRODUCTION

Free radicals are highly reactive species produced in the body during normal metabolic function or introduced from the environment. These are atoms or groups of atoms that have at least one unpaired electron, which makes them highly reactive. Reactive oxygen species (ROS) react with free radicals to become radicals themselves. Oxygen, although essential to life, is the source of the potentially damaging free radicals. Antioxidants counteract these cellular by-products, called free radicals, and bind them before they can cause damage. In fact, free radicals are believed to play a role in more than 60 different health conditions, including the aging process, cancer, and atherosclerosis.[1] Exogenous sources of free radicals include tobacco smoke, ionizing radiation, certain pollutants, organic solvents, and pesticides. Therefore, ROS can cause lipid peroxidation in foods leading to their deterioration. In addition, these ROS can easily initiate the peroxidation of membrane lipids leading to the accumulation of lipid peroxidation.[2] As a result of this, much attention has been focused on the use of antioxidants, especially natural antioxidants to inhibit lipid peroxidation and to protect from damage due to free radicals. A great number of aromatic and other medicinal plants contain chemical compounds that exhibit antioxidant properties. Sources of natural antioxidants are primarily plant phenolics that may occur in all parts of plants such as fruits, vegetables, nuts, seeds, leaves, root, and barks.

*Caesalpinia decapetala* (Roth) Alston (Caesalpiniaceae) is a thorny climber or shrub up to 25 m in height, commonly found wild in the sub-Himalayan tract and planted in hedges throughout India. It is planted in garden for its large racemes of bright yellow flowers. It is an excellent hedge-plant. A bath with decoction of the plant is useful in treatment of jaundice. The leaves are used in treatment of burns, biliousness, and stomach disorder.[3] It is used as laxative, tonic, carminative, and antipyretic.[4] Leaves and root of *C. decapetala* act as a purgative and emmenagoge.[5] The leaves of *C. decapetala* contain cassane diterpenoid, caesaldecan, spathulenol, 4, 5-epoxy-8(14)-caryophyllene, squalene, lupeol, transresveratrol, quercetin, astragalin, and stigmasterol.[6] Seven compounds were isolated and elucidated as lupeol acetate, lupeol, oleanoic acid, pentacosanoic acid 2,3-dihydroxypropyl ester, 1-(26-hydroxyhexacosanoyl)-glycerol, stigmasterol, and beta-sitosterol.[7] A new cassane diterpenoid, caesaljapin, was isolated from *C. decapetala*.[8] In Maharashtra and south India, the bark is used for tanning. In this study, we present the results of the antioxidant activities of methanol extracts of wood and pericarp of *C. decapetala*. The finding from this work may add to the overall value of the medicinal potential of the herb.

## MATERIALS AND METHODS

The wood and pericarp of *Caesalpinia decapetala* was collected from Nashik, Maharashtra, India. The plant was authenticated by Mr. P. G. Diwakar, Botanical Survey of India, Pune (Voucher no. CRP-1) and preserved in the herbarium of the department. The dried wood and pericarp (100 g) was extracted with 95% methanol for 48 h in the soxhlet apparatus. The extracts were filtered and concentrated to vacuum under reduced pressure in rotary evaporator and dried in desiccators.

### Total phenolic content

The total phenolic content of methanol extracts of *C. decapetala* wood and pericarp was determined by using the Folin-Ciocalteu assay.[9] A stock solution (1 mg/ml) of the extracts was prepared in methanol. From the stock solution, 1 ml of the extracts of different concentrations ranging from 20 to 100 μg/ml was taken into a 25 ml volumetric flask and 10 ml of water and 1.5 ml of Folin-Ciocalteau reagent were added to it. The mixture was kept for 5 min, and then 4 ml of 20% sodium carbonate solution was added and made up to 25 ml with double-distilled water. The absorbance was recorded at 765 nm after 90 min. Percentage of total phenolics was calculated from calibration curve of gallic acid plotted by using the above procedure, and expressed μg of gallic acid equivalent.

### Evaluation of free radical scavenging activity

The antioxidant activity of extracts of wood and pericarp was studied with different concentrations ranging from 100 to 1500 μg/ml. *In vitro* methods (DPPH, nitric oxide and superoxide scavenging) were used to screen the extracts for antioxidant activity. Gallic acid was used as positive control.[10]

### DPPH radical scavenging method

The stock solutions of methanolic extracts (50 μg/ml) of *C. decapetala* wood and pericarp were prepared by dissolving extracts in methanol and water. From stock solution, different concentrations in range from 100 to 1500 μg/ml were prepared and then 0.075 ml of DPPH was added in each test tube of different extracts and volume was made up to 3 ml with methanol. Tubes were allowed to rest for 15 min and the absorbance was recorded at 516 nm.[11] The decrease in absorbance with blank was also recorded. Experiment was repeated three times.

### Scavenging of nitric oxide

Different concentrations ranging from 100 to 1500 μg/ml of methanolic extract of wood and pericarp were prepared. To the extract, 1 ml of sodium nitroprusside (10 mM) was added and volumes were made up to 5 ml with methanol and incubated at room temperature for 150 min. The same reaction without the Greiss reagent, but equivalent amount of methanol served as control. After 150 min, 0.5 ml of Greiss reagent (1% sulphanilamide, 2% H_3_PO_4_ and 0.1% naphthylene dihydrochloride) was added. The absorbance was recorded at 546 nm.[12] Experiment was repeated for three times.

### Scavenging of superoxide radical

The assay was based on the capacity of the extract to inhibit formazon formation by scavenging superoxide radicals generated in the riboflavin-light-NBT system.[13] The reaction mixture contained 50 mM phosphate buffer, pH 7.6, 20 μg riboflavin, 12 mM EDTA and NBT 0.1 mg/3 ml, added in that sequence. Reaction was started by illuminating the reaction mixture containing different concentrations of sample extract for 90 s and the absorbance was measured immediately at 590 nm. Gallic acid was used as positive control.

### Lipid peroxidation assay

The degree of lipid peroxidation was evaluated by estimating the thiobarbituric acid reactive substances (TBARS) using the standard method after minor modifications.[14] Briefly, different concentrations of the extract (100-1500 μg/ml) were added to the liver homogenate. Lipid peroxidation was initiated by adding 100 μl of 15 mmol FeSO_4_ solution to 3 ml of liver homogenate (final concentration of FeSO_4_ was 0.5 mmol/ml). After 30 min, 100 μl of this reaction mixture was placed in a tube containing 1.5 ml of 10% trichloroacetic acid (TCA) and centrifuged after 10 min. The supernatant was separated and mixed with 1.5 ml of 0.67% thiobarbituric acid (TBA) in 50% acetic acid. The mixture was heated in a 100°C water bath at 85°C for 30 min to complete the reaction. The intensity of the pink colored complex was measured at 535 nm in a spectrophotometer. The TBAR were evaluated from the standard curve (absorption against concentration of tetrathoxypropane) and expressed as mmol TBARS per mg of protein. Data were expressed as Mean ± SEM, n=3.

## RESULTS AND DISCUSSION

Total phenolics and flavonoids contents results obtained in the present study revealed that the level of these phenolics compounds in the methanol extracts of the wood and pericarp of *C. decapetala* were considerable [Table 1]. Polyphenolic compounds are known to have antioxidant activity and it is likely that the activity of the extracts is due to these compounds.[15] This activity is believed to be mainly due to their redox properties, which play an important role in adsorbing and neutralizing free radicals, quenching single and triplet oxygen, or decomposing peroxides. The results strongly suggest that phenolics are important components of this plant, and some of its pharmacological effects could be attribute to the presence of these valuable constituents.

**Table 1:** Polyphenol contents of the methanol extracts of the wood and pericarp of *Caesalpinia decapetala* (*n* = 3 Mean ± SEM)

Phenolics	Wood	Pericarp
Total polyphenol^a^	13.28 ±.0057	12.68 ±.005
Total flavonoids^b^	3.93 ±.005	5.26 ±.005

a Expressed as mg gallic acid/g of dry plant material

b Expressed as mg quercetin/g of dry plant material; Values are expressed as Mean ± SEM, *n* = 3

### DPPH radical scavenging

**Figure 1:**
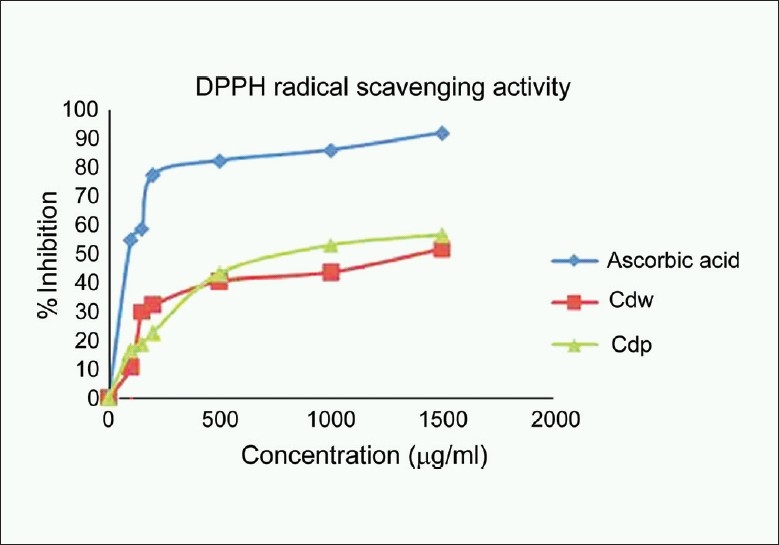
DPPH scavenging activity of the methanol extracts of wood and pericarp of *Caesalpinia decapetala*; Values are expressed as Mean ± SEM, *n* = 3

DPPH radical is commonly used as a substrate to evaluate antioxidant activity; it is a stable free radical that can accept an electron to become a stable molecule. The reduction of DPPH radical was determined by the decrease in its absorbance at 516 nm induced by antioxidants.[16] Figure 1 shows that the dose-response curve of DPPH radical scavenging activity of the methanol extract of the pericarp of *C. decapetala* had higher activity than that of wood. At a concentration of 1500 μg/ml, the scavenging activity of methanol extract of wood reached 51.65%, while at the same concentration, that of pericarp was 56.59%. Though the DPPH radical scavenging abilities of the extracts were less than those of ascorbic acid (91.62%) at1500 μg/ ml, the study showed that the extracts have the proton-donating ability and could serve as free radical inhibitor or scavengers, acting possibly as primary antioxidants.

### Superoxide anion radical scavenging

**Figure 2:**
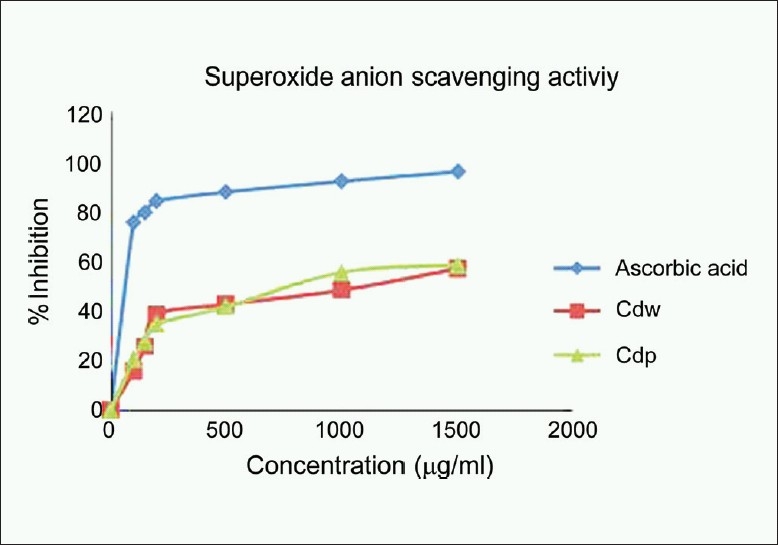
Superoxide anion radical scavenging activity of the methanol extracts of wood and pericarp of *Caesalpinia decapetala*; Values are expressed as Mean ± SEM, *n* = 3

Superoxide anion is a reduced from of molecular oxygen, by receiving an electron. It is also an initial free radical formed from mitochondrial electron transport system. Mitochondria generate energy using four electron chain reactions, reducing oxygen to water. Some of the electrons escaping from the chain reaction of mitochondria directly react with oxygen and from superoxide anion.[17] The results are shown in Figure 2. *C.decapetala* wood and pericarp had a significant scavenging activity on the superoxide anion radicals in a dose-dependent manner compared with ascorbic acid.

### Nitric oxide scavenging assay

**Figure 3:**
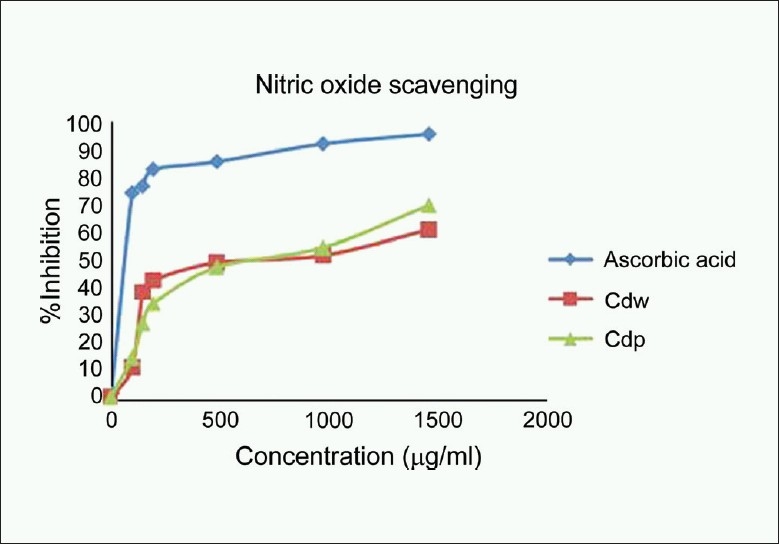
Nitric oxide scavenging activity of the methanol extracts of wood and pericarp of *Caesalpinia decapetala*; Values are expressed as Mean ± SEM, *n* = 3

Nitric oxide scavenging effect of wood and pericarp of *C. decapetala* was found to be 60.67% and 69.65% at 1500 μg/ml respectively which was compared with ascorbic acid (95.12% at 1500 μg/ml). Nitric oxide (NO) is an important chemical mediator generated by endothelial cells, macrophages, neurons, etc. and involved in the regulation of various physiological processes. Excess concentration of NO is associated with several diseases. Oxygen reacts with the excess nitric oxide to generate nitrite and peroxynitrite anions, which act as free radicals.[18] In the present study, the *C. decapetala* competes with oxygen to react with nitric oxide and thus inhibit generation of anion [Figure 3].

### Lipid peroxidation assay

**Figure 4:**
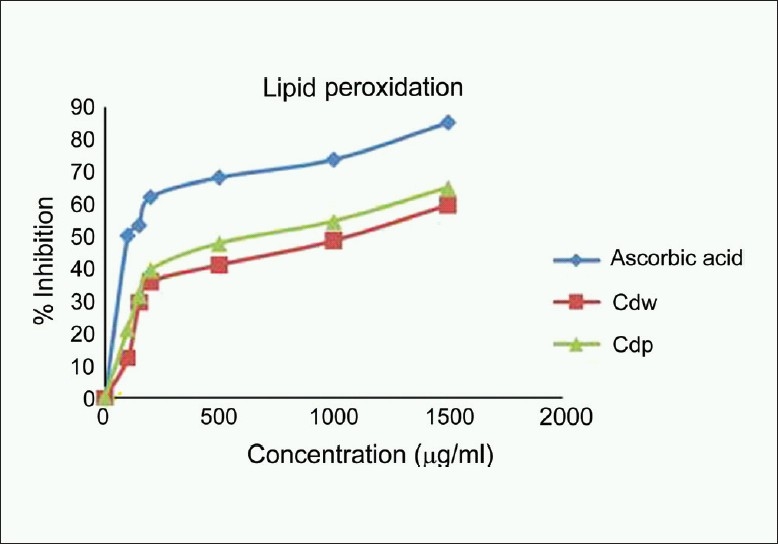
Lipid peroxidation scavenging activity of the methanol extracts of wood and pericarp of *Caesalpinia decapetala*; Values are expressed as Mean ± SEM, *n* = 3

*C. decapetala* wood and pericarp extracts caused a dose-dependent protection against lipid peroxidation with 59.87% and 65.18% protection respectively at 1500 μg/ml concentration. Inhibition of lipid peroxidation by ferrous sulfate take place either through ferryl perferryl complex or through OH radical. Ferryl-perferryl complex can also initiate lipid peroxidation on its own in a similar manner as OH, though it is less reactive than OH. In iron-induced lipid peroxidation, however, the role of OH was not found to be significant. The values of TBARS upon incubation with the extract are represented in Figure 4. The inhibition could be caused by the absence of ferryl-perferryl complex or by scavenging of OH radical or superoxide radical or by changing the Fe^3+^ /Fe^2+^ ration or by reducing the rate of conversion of ferrousd to ferric ion or by chelating the iron itself.[19]
